# Correction: ‘Digital micro-banking as a health and protection intervention for street-connected children and youth? Analysis from a Togolese pilot’

**DOI:** 10.1136/bmjpo-2023-002423corr1

**Published:** 2024-09-10

 Howard N. Digital micro-banking as a health and protection intervention for street-connected children and youth? Analysis from a Togolese pilot. *BMJ Paediatrics Open* 2024;8:e002423. doi:10.1136/bmjpo-2023-002423

This article has been corrected since it was published online. The initial ethics statement attached to this article included reference to the University of Bath’s Social Science Ethics Committee in error. This has been removed and the Committee is aware.

