# Supplementary material for: Digital micro-banking as a health and protection intervention for street-connected children and youth? Analysis from a Togolese pilot
Source: BMJ Paediatr Open. 2024 Apr 30;8(1):e002423. doi: 10.1136/bmjpo-2023-002423 (PMC11086537; doi:10.1136/bmjpo-2023-002423)
Supplement: Author's manuscript [file bmjpo-2023-002423.draft_revisions.pdf]

BMJ Paediatrics Open

BMJ Paediatrics Open is committed to open peer review. As part of this commitment we make the peer review history of every article we publish publicly available.

When an article is published we post the peer reviewers' comments and the authors' responses online. We also post the versions of the paper that were used during peer review. These are the versions that the peer review comments apply to.

The versions of the paper that follow are the versions that were submitted during the peer review process. They are not the versions of record or the final published versions. They should not be cited or distributed as the published version of this manuscript.

BMJ Paediatrics Open is an open access journal and the full, final, typeset and author-corrected version of record of the manuscript is available on our site with no access controls, subscription charges or pay-per-view fees (http://bmjpaedsopen.bmj.com).

If you have any questions on BMJ Paediatrics Open's open peer review process please email info.bmjpo@bmj.com

BMJ Paediatrics Open

Digital Micro-Banking as a Health and Protection Intervention for Street-Connected Children and Youth? Analysis from a Togolese Pilot

(For consideration in the Special Collection on Health and Wellbeing of Street and Working Children, in collaboration with the International Society for Social Pediatrics and Child Health (ISSOP))

Journal:	*BMJ Paediatrics Open*
Manuscript ID	bmjpo-2023-002423
Article Type:	Original research
Date Submitted by the Author:	23-Dec-2023
Complete List of Authors:	Howard, Neil; University of Bath Faculty of Humanities and Social Sciences, Social
Keywords:	Anthropology, Adolescent Health, Child Abuse, Social work

SCHOLARONE™
Manuscripts

I, the Submitting Author has the right to grant and does grant on behalf of all authors of the Work (as defined in the below author licence), an exclusive licence and/or a non-exclusive licence for contributions from authors who are: i) UK Crown employees; ii) where BMJ has agreed a CC-BY licence shall apply, and/or iii) in accordance with the terms applicable for US Federal Government officers or employees acting as part of their official duties; on a worldwide, perpetual, irrevocable, royalty-free basis to BMJ Publishing Group Ltd ("BMJ") its licensees and where the relevant Journal is co-owned by BMJ to the co-owners of the Journal, to publish the Work in this journal and any other BMJ products and to exploit all rights, as set out in our licence.

The Submitting Author accepts and understands that any supply made under these terms is made by BMJ to the Submitting Author unless you are acting as an employee on behalf of your employer or a postgraduate student of an affiliated institution which is paying any applicable article publishing charge ("APC") for Open Access articles. Where the Submitting Author wishes to make the Work available on an Open Access basis (and intends to pay the relevant APC), the terms of reuse of such Open Access shall be governed by a Creative Commons licence – details of these licences and which Creative Commons licence will apply to this Work are set out in our licence referred to above.

Other than as permitted in any relevant BMJ Author's Self Archiving Policies, I confirm this Work has not been accepted for publication elsewhere, is not being considered for publication elsewhere and does not duplicate material already published. I confirm all authors consent to publication of this Work and authorise the granting of this licence.

Confidential: For Review Only

Title Page

Title

Digital Micro-Banking as a Health and Protection Intervention for Street-Connected Children and Youth? Analysis from a Togolese Pilot

Corresponding Author

Neil Howard, PhD,
University of Bath
Claverton Down
BA2 7AY
UK
Email: n.p.howard@bath.ac.uk

Key Words

Street-connected children and youth, child protection, violence against children, child banking

Word Count

2268

Acknowledgement

The author would like to acknowledge the contributions of the entire SaVa team to the design and evaluation of the project.

Contributorship Statement

Dr. Howard is the sole author of this article.

Funding Statement

This work was supported by an Innovation Fund grant (Safe Savings – SaVa') from the international child protection organisation, Terre des Hommes Lausanne (Tdh): Avenue de Montchoisi 15, CH-1006, Lausanne, Switzerland. It partnered Tdh, Ecobank, the African Movement of Working Children and Youth (AMWCY), and the University of Bath (UoB).

Confidential: For Review Only

Confidential: For Review Only

Ethics approval statements

This project was granted ethical approval after internal review by Tdh's project ethics advisor on Dec 1 2018. It was also approved by the University of Bath's Social Sciences Research Ethics Committee, with reference number 2670-2808.

Competing Interests Statement

The author completed the ICMJE uniform disclosure form at http://www.icmje.org/disclosure-of-interest/ and declares: I received funding from Terre des Hommes to lead the team conducting the action research that led to the proposal of the pilot studied in this paper; I further received funding to participate in design and evaluation of the pilot. This funding ended in 2020.

Patient and Public Involvement Statement

Members of the public were involved as research participants at every stage of the project reported on. The intervention design was rooted in a participatory action research project that consulted some of the children and youth (C&Y) who would eventually participate in the intervention and its evaluation (cited below as Howard et al. 2018). C&Y researchers were recruited and trained to gather and analyse data; initial findings were shared and cross-checked with them. Interviews with C&Y were open-ended and left space for C&Y to guide them. Fun, creative methods were also used.

Data Sharing Statement

The unpublished report behind this paper can be freely downloaded at https://purehost.bath.ac.uk/ws/portalfiles/portal/212996491/_SaVa_Pilot_Evaluation_Report.pdf. Interview schedules and anonymised primary data will be shared upon request.

Main Document

Abstract

Background:

The SaVa pilot offered street-connected children and youth (C&Y) access to digital micro-banking services to help them save money and thus avoid the violence associated with theft at night. It further used the micro-banking service as a gateway to attract C&Y towards other health and protection services. It took place over 12 months in Lomé, Togo, and involved one social worker (SW) and a group of volunteers deployed in an NGO-funded drop-in centre which served a community of street-connected C&Y by the Aflao border with Ghana.

Methods:

Research involved C&Y researchers trained to interview and use collective drawing with their peers, which they did on a bi-monthly basis. The SW recorded information relating to case management, training, and additional services offered. He also administered a survey to all C&Y at the point of entry into the SaVa project and after 1 year, asking basic questions about things like recent experiences of violence or theft. At the end of the pilot's 12 months, the author conducted interviews with C&Y using the bank (6), C&Y researchers who used the bank (2), project staff and steering committee partners (10), and child protection stakeholders (4). One focus group was also conducted with C&Y participants (10), alongside limited participant observation of where C&Y sleep and work.

Results:

Findings suggest that the intervention was highly successful in encouraging children to open accounts, to save money, and in contributing to a reduction in both theft and associated violence. The project further supported the delivery of significant additional services, such as counselling, school reinsertion, or removal from the streets.

Conclusions:

Analysis suggests not only that it is possible to offer street-connected C&Y financial services but that that the offer of micro-banking has significant potential as a health and protection intervention, especially in combination with the provision of a safe space, dedicated SW support, and add-on services. International child protection organisations are encouraged to include digital micro-banking services as part of their suite of offerings to street-connected C&Y.

Key Findings

Confidential: For Review Only

There are millions of street-connected C&Y all around the world and they are known to be vulnerable to theft and associated violence. Lacking access to formal financial services, they typically rely on informal and often unreliable savings mechanisms which deepen their vulnerability. This study shows that with the right institutional accompaniment, digital micro-banking is possible for street-connected C&Y and that it can contribute to increased savings and reduced theft and associated violence. It further shows that digital micro-banking can be used as an attractive 'hook' to bring C&W into the orbit of additional services. The findings strongly suggest that child protection actors should consider incorporating the offer of digital micro-banking services alongside more traditional offers of safe spaces, SW support, and service referral.

Introduction

There are millions of street-connected C&Y around the world and they are known to be vulnerable to violence, including theft and associated assault [1]. In 2017, the author led a participatory action research study with street-connected migrant and working C&Y in five West African cities, which found that theft, especially at night, was among the greatest issues they faced [2]. Not only was theft traumatic and often accompanied by violence, but the fact that C&Y were so frequently robbed meant that they were effectively stuck in a poverty trap, unable to accumulate enough money to get off the streets or to go home. The SaVa pilot emerged from this study and was implemented by the international child protection organisation, Terre des Hommes (Tdh). It sought to examine the theft- and violence-reduction potential of providing these street-connected C&Y with access to digital micro-banking services in combination with more 'traditional' support such as a safe space, SW accompaniment, and referral. The hypothesis was that if C&Y had somewhere safe and accessible to put their money, they would be less likely to keep it on their person and thus less vulnerable to theft. We also anticipated that micro-banking services would act as a 'gateway' for additional child protection services on offer. The rest of this paper reports the pilot's findings and discusses their implications[1].

Methods

The SaVa model was straightforward. Operationally, it centred on the Tdh *Hope Point* at the Aflao border with Ghana. *Hope Points* are safe spaces for C&Y to come and rest, play, or receive SW support. This one served the street-connected population based around Lomé beach and was staffed by a senior SW. He was supported on a rotating basis by four experienced activists from the African Movement for Working Children and Youth (AMWCY), who did outreach work and contributed to the management of micro-banking services.

Image 1: The Hope Point Drop-In Centre

[1] Parts of this paper draw on and reproduce text from the project's evaluation [3].

Confidential: For Review Only

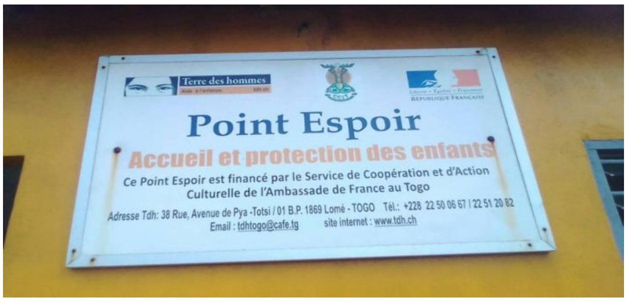

© Author

Day-to-day, the *Hope Point* operated like a bank, with C&Y coming to deposit and withdraw their money during opening hours. Banking was provided by Ecobank in the form of an *Xpress Point*, which is an Ecobank-approved, sub-contracted mobile money service. This bank included a counter, secured smartphones for transactions, and SIM cards for each individual account, which were kept in the *Hope Point* for safety overnight. Crucially, while Ecobank provided the financial infrastructure, Tdh acted as the legal sponsor for each account – in other words, each mobile money account and the SIM card associated with it was opened in Tdh's name (so as to enable the project and the bank to comply with legal KYC requirements), but Tdh kept a ledger in the *Hope Point* linking each SIM to their eventual C&Y 'owner'. This was a significant innovation. Through it, the project aimed to open 150 accounts in its first year and in turn to assess whether these prompted a reduction in the incidence of violence and theft. The SW, AMWCY activists, and Ecobank staff offered basic financial literacy training to interested C&Y, while the *Hope Point* functioned as a place of refuge. Although the *Hope Point* was already known to and used by C&Y, it was hoped that SaVa could attract more of them to it, with the bank serving as an entry point for orientation towards other necessary services (such as healthcare or, for the youngest, school reinsertion).

Research into the pilot sought to understand whether, how, and for whom the project 'worked', with which implications for child protection practice. Methods were mixed but lack the representativity of a Randomised Control Trial. C&Y researchers were trained to interview and use collective drawing with their peers, which they did on a bi-monthly basis. The SW recorded information relating to case management, training, and additional services offered. He also administered a survey to all C&Y at the point of entry into the SaVa project and after 1 year, asking basic questions such as around recent experiences of violence or theft. At the end of the pilot's 12 months, the author conducted interviews with C&Y using the bank (6), C&Y researchers who used the bank (2), project staff and steering committee partners (10), and child protection stakeholders (4). One focus group was also conducted with C&Y participants (10), alongside limited participant observation of where C&Y sleep and work. This broad dataset offers a solid base from which to interpret the project's findings. Full discussion of the project and its research methods can be found and freely accessed in the open-access evaluation [3].

Results

Financial Matters

Confidential: For Review Only

Consultations with Togolese child protection actors established a target of working with 150 C&Y over the year of the pilot. In terms of accounts opened, this was exceeded by over 30%, with 200 accounts opened. Total deposits surpassed 600,000 FCFA, close to 800 GBP, with withdrawals totalling around 440,000 FCFA. Almost 1,400 transactions took place, averaging around 400 FCFA per transaction, which is more than most C&Y spend on food in a day.

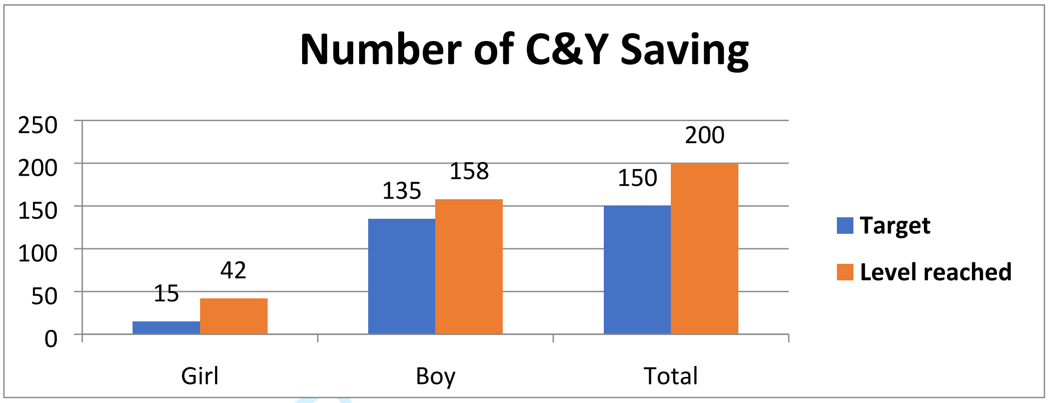

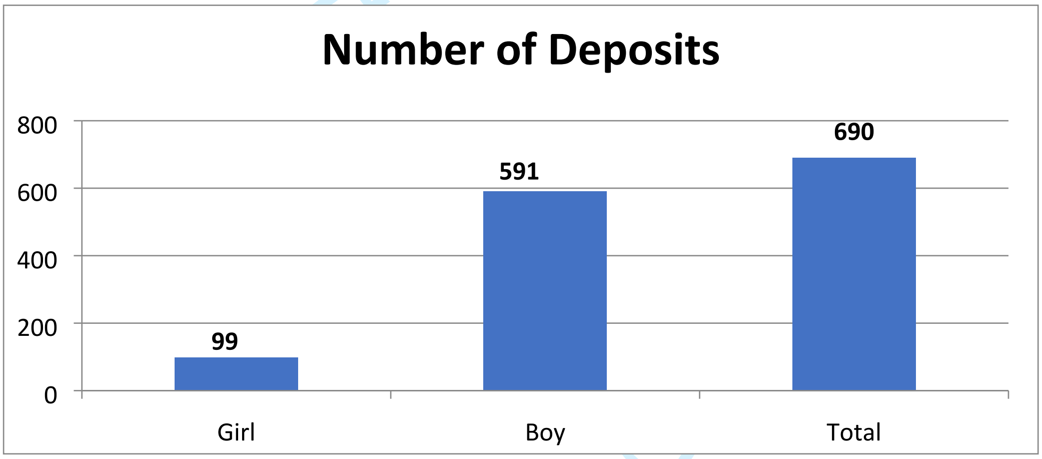

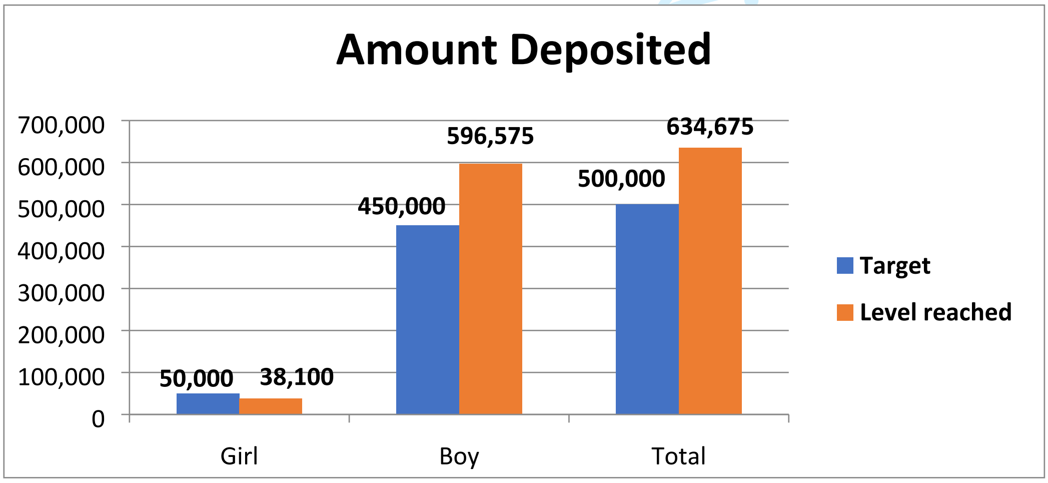

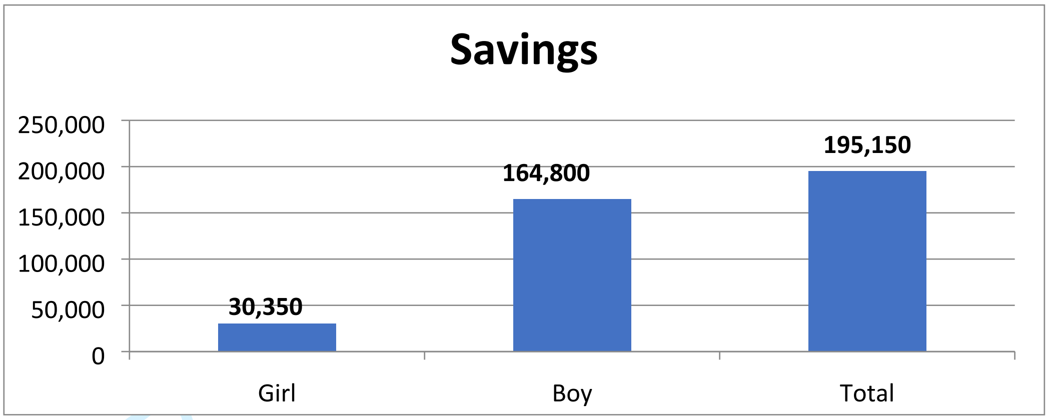

Although excitement was palpable at the existence of a bank ‘just for them’, C&W reported numerous difficulties in using it. Opening times – which were restricted to daylight hours – were often inconvenient, especially for C&W busy in the day or making their money and wishing to deposit it at night. The lack of reliable internet access – on which the banking app depends – hampered various financial transactions. And the app itself, at the time relatively new, often crashed, or experienced issues that prevented the processing of transactions.

These obstacles make the project’s positive financial numbers appear even more impressive. Qualitative data suggest that they owe much to the efforts of the SW, the volunteers, and the Ecobank staff, who conducted awareness-raising activities to promote the bank in the immediate vicinity of the *Hope Point* and further afield where street-connected, migrant and working C&Y were known to be concentrated. Football matches and music were also staged, whilst the promise of a (much-needed) free meal brought many in in the early days.

A Reduction in Violence

C&Y using the bank reported a drastic reduction in theft and violence over the course of the project’s pilot year. Fully 100% of the C&Y involved in the project reported experiencing some form of recent violence when opening their accounts at the start. At the end of the 12 pilot months, this figure was down below 10% – a 90% reduction. Theft similarly plummeted. At the project’s inception, the vast majority of participating C&Y reported having been robbed at some point over the preceding 6 months, often more than once. By contrast, at the end of the pilot period, 67% of respondents had been be ‘theft-free’ for the past 6 months.

Confidential: For Review Only

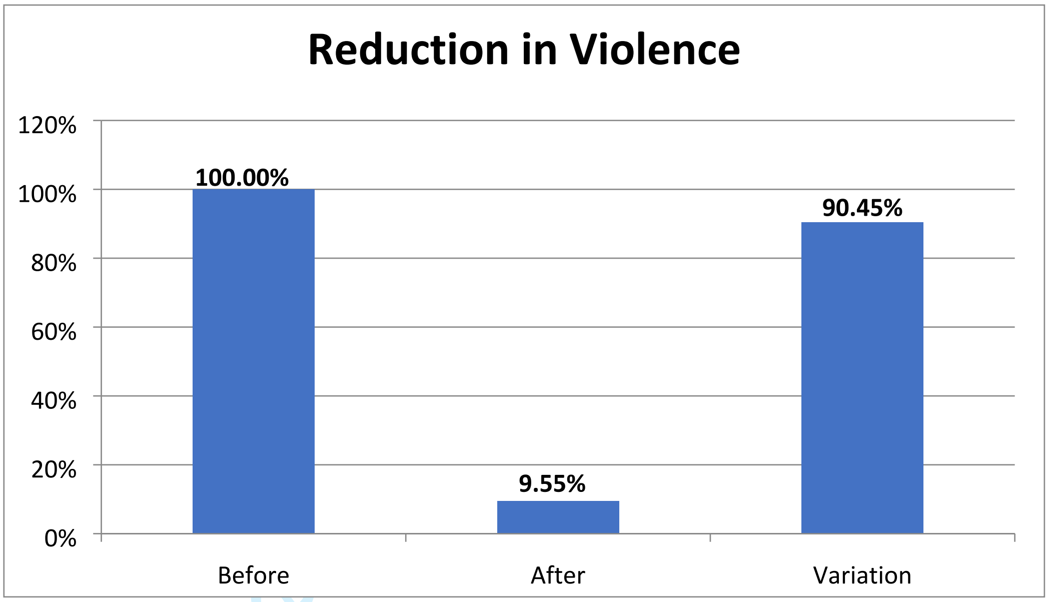

What explains these reductions? Scientifically, it is possible that they are just chance. There is also a risk of respondent bias, with C&Y answering survey or interview questions with what they think those asking would like to hear. Yet qualitative research point squarely in the direction of alternative explanations. First, as anticipated, those depositing their money in the digital micro-bank became less attractive to potential thieves and claimed as much themselves. Second, having a savings account reduced the imperative for C&Y to quickly spend what money they have, which is important because when they burn through cash for fear of loss, they end up with nothing and thus themselves resort to theft in order to be able to eat. Third, and most importantly, data strongly suggest that the sensitisation and diligent on-the-ground work by the SW and volunteers were paramount in shifting social norms away from an acceptance of theft and violence. In this respect, digital micro-banking represented a kind of 'hook' around which more traditional behaviour change work could take place.

Skills Development and Hope

The opening of individual savings accounts presented the SW and *Hope Point* volunteers with an opportunity to offer C&Y trainings in financial literacy, numeracy, and budgeting. These were complimented by guidance on how to develop 'a life plan'. Data suggest that this orientation was positively experienced by C&Y, many of whom reported the project giving them skills, 'a sense of hope', and 'something to work for'. Importantly, the psychological function of saving appears to have mattered for many, in that it gave C&Y an anchor in the future, something safe to work towards or through, and thus a vehicle for their aspiration. Many translated this aspiration into plans to accumulate enough savings to enter an apprenticeship. Once again, though, the impact of digital micro-banking cannot be disentangled from the impact of professional psycho-social support in the form of the skilled SW or the provision of a safe space in which the C&Y could gather. Indeed, interview transcripts suggest that for some C&Y, the *Hope Point* itself was the most important thing the project offered, since it represented a place of refuge in lives that are hard, insecure, and often vulnerable:

'Here is somewhere we can come and rest, sleep, be safe…'
Interview with a 15-year-old Boy

'Here we can play, eat, hang out with our friends…'
15-year-old male Focus Group Participant

A Gateway for Other Services

As anticipated, the offer of digital micro-banking services to these C&Y acted as an entry point for providing other support and services. 54 C&Y, for example, were oriented towards social or health services according to their needs. Many benefited from interpersonal or family mediation. Some received empathy when they came to the *Hope Point* with their difficulties; others advice when they needed it. As the table below makes clear, fully 111 C&Y went through situation-changing interventions during the life of the pilot, including 33 leaving the streets.

C&W SUPPORTED THROUGH HOPE POINT			
**Target Group**	**Boys**	**Girls**	**Total**
Children put back in school	19	0	19
Children kept in school	08	07	15
Children taken out of work and put in an apprenticeship	13	06	19
Children supported to stay in an apprenticeship	02	06	08
Children taken off the streets and reunited with their families	33	17	50
**Totals**	**75**	**36**	**111**

Importantly, these services constitute the core offerings associated with the *Hope Point* and its operation; it is difficult therefore to establish whether and to what extent the addition of micro-banking services contributed to any increase in numbers.

Discussion

Evaluation of the SaVa pilot demonstrates, first, that it is possible to provide financial inclusion for street-connected C&Y – a population almost universally excluded from formal financial services. The SaVa model of partnering a bank and a child protection institution willing to act as the 'sponsor' complying with KYC legislation and then offering over-the-counter services is unique. Although there is some precedent for C&Y savings groups in Africa [4-8] and for pseudo-banking services offered to street-connected C&Y in South Asia [9-10], the project's benchmarking study found no evidence anywhere either of the widespread provision of formal financial services to street-connected C&Y or of the use of digital services to this end [11]. In this respect, SaVa represents a potentially significant departure for C&Y financial inclusion, in particular for the highly vulnerable, typically marginalised group that is the street-connected.

Confidential: For Review Only

In addition, the study shows that, with the right supportive accompaniment, digital micro-banking can contribute to increased financial security and reduced theft and associated violence for street-connected C&Y. Although the evaluation methodology does not enable full disaggregation of the relative impacts of SW support, the provision of a safe space, and the provision of banking services, we can nevertheless be highly confident that this *combination* is effective. Indeed, perhaps the major conclusion of our analysis must be that this combination 'works' for the vulnerable population in question. C&Y enjoyed having access to financial services and opening a savings account evidently supported the lengthening of their financial time horizons, which in turn had consequences for their ability to plan and aspire as well as to avoid quick spending and thus future theft. C&Y further enjoyed the safety and succour available at the *Hope Point* and the support provided by their SW, which included orientation to parallel services. In this respect, the study confirms that 'traditional' offers such as safe spaces and orientation to other services must continue to play a role in C&Y protection, but that digital micro-banking is a worthy addition to the protective mix. It further suggests that the effectiveness of digital micro-banking will depend on its being embedded in a traditional C&Y protection offering.

This study is evidently only a single case and a pilot at that; as such, its findings are necessarily limited. This is compounded by the fact that evaluation was not through an RCT, meaning that generalisability is impossible. Nevertheless, the findings are intriguing enough to suggest that replication and scale-up is worthwhile for the international child protection community, and that policy-makers and practitioners would do well to replicate the SaVa model and conduct further research towards refinement.

References

[1] Committee on the Rights of the Child (CRC) (2017), *General comment No. 21 (2017) on children in street situations*, Geneva: Office for the Commission on Human Rights. Available at: https://tbinternet.ohchr.org/_layouts/15/treatybodyexternal/Download.aspx?symbolno=CRC%2fC%2fGC%2f21&Lang=en

[2] Howard, N., Jacquemin, M. & Thorsen, D. (2018), *Protecting Child Migrants Along the Abidjan-Lagos Corridor: Baseline Research Report*, Lausanne: Terre des Hommes Foundation. Available at: https://purehost.bath.ac.uk/ws/portalfiles/portal/203723196/26.pdf

[3] Howard, N. (2020), *Pilot Evaluation Report: Sava - Safe Saving*, Lausanne: Terre des Hommes Foundation. Available at: https://purehost.bath.ac.uk/ws/portalfiles/portal/212996491/_SaVa_Pilot_Evaluation_Report.pdf

[4] Deshpande, R. and Zimmerman, J., 2010, *Youth Savings in Developing Countries: Trends in Practice, Gaps in Knowledge*, YouthSave Consortium, Washington D.C.

Confidential: For Review Only

[5] Zou, L., Tlapek, S., Njenga, G., Appiah, E., Opai-Tetteh, D. and Sherraden, M., (2015), 'Facilitators and Obstacles in Youth Saving: Perspectives from Ghana and Kenya', *Global Social Welfare*, 2.2: pp. 65–74.

[6] Meagher, K., (2016), Conference Report, *Consolidating Africa's Mobile Banking Revolution*, Blavatnik School of Public Policy, University of Oxford.

[7] Flynn, J. and Sumberg, J., (2017), *Youth Savings Groups in Africa: They're a Family Affair*, Enterprise Development and Microfinance, 09/2017, Vol.28(3), pp. 147-161.

[8] Flynn, J. and Sumberg, J., (2018), *Savings Groups for Young Africans: A (Financial) Game Changer*?**,** Opinion, Institute of Development Studies (IDS). Accessible at: http://www.ids.ac.uk/opinion/savings-groups-for-young-africans-a-financial-game-changer.

[9] Butterflies (2017), *My Khazana, My Dreams: A Book on Children's Experiences with Children's Development Khazana*, July 2017, New Delhi: Butterflies NGO.

[10] Yiga, D. and Raman, V., (2017), *Final Evaluation Report: Butterflies Investment Grant 2011-2017*, Kampala: Development Links Consulting Ltd.

[11] Amirali, A. (2018), *Child-Focused Micro Banks: Learning from Existing Programme Models*, Unpublished Report, Lausanne: Terre des Hommes Lausanne.

Confidential: For Review Only

BMJ Paediatrics Open

Digital Micro-Banking as a Health and Protection Intervention for Street-Connected Children and Youth? Analysis from a Togolese Pilot

(For consideration in the Special Collection on Health and Wellbeing of Street and Working Children, in collaboration with the International Society for Social Pediatrics and Child Health (ISSOP))

Journal:	*BMJ Paediatrics Open*
Manuscript ID	bmjpo-2023-002423.R1
Article Type:	Original research
Date Submitted by the Author:	29-Feb-2024
Complete List of Authors:	Howard, Neil; University of Bath Faculty of Humanities and Social Sciences, Social
Keywords:	Anthropology, Adolescent Health, Child Abuse, Social work

SCHOLARONE™
Manuscripts

I, the Submitting Author has the right to grant and does grant on behalf of all authors of the Work (as defined in the below author licence), an exclusive licence and/or a non-exclusive licence for contributions from authors who are: i) UK Crown employees; ii) where BMJ has agreed a CC-BY licence shall apply, and/or iii) in accordance with the terms applicable for US Federal Government officers or employees acting as part of their official duties; on a worldwide, perpetual, irrevocable, royalty-free basis to BMJ Publishing Group Ltd ("BMJ") its licensees and where the relevant Journal is co-owned by BMJ to the co-owners of the Journal, to publish the Work in this journal and any other BMJ products and to exploit all rights, as set out in our licence.

The Submitting Author accepts and understands that any supply made under these terms is made by BMJ to the Submitting Author unless you are acting as an employee on behalf of your employer or a postgraduate student of an affiliated institution which is paying any applicable article publishing charge ("APC") for Open Access articles. Where the Submitting Author wishes to make the Work available on an Open Access basis (and intends to pay the relevant APC), the terms of reuse of such Open Access shall be governed by a Creative Commons licence – details of these licences and which Creative Commons licence will apply to this Work are set out in our licence referred to above.

Other than as permitted in any relevant BMJ Author's Self Archiving Policies, I confirm this Work has not been accepted for publication elsewhere, is not being considered for publication elsewhere and does not duplicate material already published. I confirm all authors consent to publication of this Work and authorise the granting of this licence.

Confidential: For Review Only

Title Page

Title

Digital Micro-Banking as a Health and Protection Intervention for Street-Connected Children and Youth? Analysis from a Togolese Pilot

Corresponding Author

Neil Howard, PhD,
University of Bath
Claverton Down
BA2 7AY
UK
Email: nph28@bath.ac.uk

Key Words

Street-connected children and youth, child protection, violence against children, child banking

Word Count

2495

Acknowledgement

The author would like to acknowledge the contributions of the entire SaVa project team to the design and evaluation of the project.

Contributorship Statement

Dr. Howard is the sole author of this article.

Funding Statement

This work was supported by an Innovation Fund grant (Safe Savings – SaVa') from the international child protection organisation, Terre des Hommes Lausanne (Tdh): Avenue de Montchoisi 15, CH-1006, Lausanne, Switzerland. It partnered Tdh, Ecobank, the African Movement of Working Children and Youth (AMWCY), and the University of Bath (UoB).

Confidential: For Review Only

Ethics approval statements

This project was granted ethical approval after internal review by Tdh's project ethics advisor on Dec 1 2018.

Competing Interests Statement

The author completed the ICMJE uniform disclosure form at http://www.icmje.org/disclosure-of-interest/ and declares: I received funding from Terre des Hommes to lead the team conducting the action research that led to the proposal of the pilot studied in this paper; I further received funding to participate in design and evaluation of the pilot. This funding ended in 2020.

Patient and Public Involvement Statement

Members of the public were involved as research participants at every stage of the project reported on. The intervention design was rooted in a participatory action research project that consulted some of the children and youth (C&Y) who would eventually participate in the intervention and its evaluation (cited below as Howard et al. 2018). C&Y researchers were recruited and trained to gather and analyse data; initial findings were shared and cross-checked with them. Interviews with C&Y were open-ended and left space for C&Y to guide them. Fun, creative methods were also used.

Data Sharing Statement

The unpublished report behind this paper can be freely downloaded at https://purehost.bath.ac.uk/ws/portalfiles/portal/212996491/_SaVa_Pilot_Evaluation_Report.pdf. Interview schedules and some anonymised primary data may be shared upon request; others cannot be shared for data protection reasons.

Confidential: For Review Only

Main Document

Abstract

Background:

The SaVa pilot offered street-connected children and youth (C&Y) access to digital micro-banking services to help them save money and thus avoid the violence associated with theft at night. It further used the micro-banking service as a gateway to attract C&Y towards other health and protection services. It took place over 12 months in Lomé, Togo, and involved one social worker (SW) and a group of volunteers running 'the bank' in an NGO-funded drop-in centre by the Aflao border with Ghana.

Methods:

C&Y peer researchers were trained to interview and use collective drawing with their peers, which they did on a bi-monthly basis. The SW recorded information about case management, training, and additional services offered. He administered a survey to all C&Y at the point of entry into the project and after 1 year, asking basic questions about things like recent experiences of violence or theft. At the end of the pilot's 12 months, the author conducted interviews with C&Y using the bank (6), C&Y researchers who used the bank (2), project staff and steering committee partners (10), and child protection stakeholders (4). One focus group was also conducted with C&Y participants (10), alongside limited participant observation of where C&Y sleep and work.

Results:

Findings suggest that the intervention was highly successful in encouraging children to open accounts, to save money, and in contributing to a reduction in both theft and associated violence. The project further supported the delivery of additional services, such as counselling, school reinsertion, or removal from the streets.

Conclusions:

Analysis suggests not only that it is possible to offer street-connected C&Y formal financial services but that that this offer has potential as a health and protection intervention, especially in combination with the provision of a safe space, dedicated SW support, and add-on services.

Key Message

- **What is already known on this topic**

Confidential: For Review Only

There are millions of street-connected C&Y all around the world and they are known to be vulnerable to theft and associated violence. Lacking access to formal financial services, they typically rely on informal and often unreliable savings mechanisms which deepen their vulnerability.

- **What this study adds**

This study shows that with the right institutional accompaniment, digital micro-banking is possible for street-connected C&Y and that it can contribute to increased savings and reduced theft and associated violence. It further shows that digital micro-banking can be used as an attractive 'hook' to bring C&Y into the orbit of additional services.

- **How this study might affect research, practice or policy**

The findings strongly suggest that child protection actors should consider incorporating the offer of digital micro-banking services alongside more traditional offers of safe spaces, SW support, and service referral.

Key Findings

There are millions of street-connected C&Y all around the world and they are known to be vulnerable to theft and associated violence. Lacking access to formal financial services, they typically rely on informal and often unreliable savings mechanisms which deepen their vulnerability. This study shows that with the right institutional accompaniment, digital micro-banking is possible for street-connected C&Y and that it can contribute to increased savings and reduced theft and associated violence. It further shows that digital micro-banking can be used as an attractive 'hook' to bring C&Y into the orbit of additional services. The findings strongly suggest that child protection actors should consider incorporating the offer of digital micro-banking services alongside more traditional offers of safe spaces, SW support, and service referral.

Introduction

There are millions of street-connected C&Y around the world and they are known to be vulnerable to violence, including theft and associated assault [1]. In 2017, the author led a participatory action research study with street-connected migrant and working C&Y in five West African cities, which found that theft, especially at night, was among the greatest issues they faced [2]. Not only was theft traumatic and often accompanied by violence, but the fact that C&Y were so frequently robbed meant that they were effectively stuck in a poverty trap, unable to accumulate enough money to get off the streets or to go home. This dynamic is well-established in the literature, which shows that C&Y use multiple, often quite ingenious, informal avenues for saving – ranging from entrusting their money to a local adult to burying it overnight – but that none of these remain reliable in the long-term [3-7]. The literature further emphasises that there are substantial legal and cultural barriers to offering street-connected C&Y formal financial services. Lacking a fixed address or formal identification documents, few street-connected C&Y can meet the legal Know-Your-Customer

Confidential: For Review Only

requirements underpinning most formal banking, including mobile money banking, while informal barriers such as discrimination on the part of bank staff or shame on the part of C&Y are also impactful [3, 8]. Although there is some precedent for C&Y savings groups in Africa [9-13] and for pseudo-banking services for street-connected C&Y in South Asia [4, 14], the project's benchmarking study found no evidence either of the widespread provision of formal financial services to street-connected C&Y or of the use of digital services to this end [3]. Consequently, C&Y remain excluded from financial services and more likely to face theft, violence, and the ongoing cycle of poverty.

The SaVa pilot emerged in this context and was implemented by the international child protection organisation, Terre des Hommes (Tdh). It sought to examine the theft- and violence-reduction potential of providing these street-connected C&Y with access to digital micro-banking services in combination with more 'traditional' support such as a safe space, SW accompaniment, and referral. The hypothesis was that if C&Y had somewhere safe and accessible to put their money, they would be less likely to keep it on their person and thus less vulnerable to theft, whilst also being less likely to avail themselves of parallel informal savings mechanisms that often let them down. We also anticipated that children would be attracted to the technology of the micro-banking service, which would in turn act as a 'gateway' for additional child protection services. Findings confirm these hypotheses and show that with the right institutional accompaniment, digital micro-banking *is* possible for street-connected C&Y and that it can contribute to increased savings and reduced theft and associated violence. They further show that digital micro-banking can be used as an attractive 'hook' to bring C&Y into the orbit of additional services. The findings suggest that child protection actors should consider incorporating this offer alongside more traditional offers of safe spaces, SW support, and service referral[1].

Methods

The SaVa model was straightforward. Operationally, it centred on the Tdh *Hope Point* at the Aflao border with Ghana. *Hope Points* are safe spaces for C&Y to come and rest, play, or receive SW support (see Figure 1 below). This one served the street-connected population based around Lomé beach and was staffed by a senior SW. He was supported on a rotating basis by four experienced activists from the African Movement for Working Children and Youth (AMWCY), who did outreach work and contributed to the management of micro-banking services.

Figure 1: The Hope Point Drop-In Centre

© Author – Permission Granted for Use

Day-to-day, the *Hope Point* operated like a bank, with C&Y coming to deposit and withdraw money during opening hours. Banking was provided by Ecobank in the form of an *Xpress Point*, which is an Ecobank-approved, sub-contracted mobile money service. This bank included a counter, secured smartphones for transactions, and SIM cards for each individual account, which were kept in the *Hope Point* overnight to prevent loss or theft. Crucially, while Ecobank

[1] Parts of this paper draw on and reproduce text from the project's evaluation [15].

Confidential: For Review Only

provided the financial infrastructure, Tdh acted as the legal sponsor for each account – in other words, each mobile money account and the SIM card associated with it was opened in Tdh's name (so as to enable the project and the bank to comply with Know-Your-Customer requirements), but Tdh kept a ledger in the *Hope Point* linking each SIM to their eventual C&Y 'owner'. This was a significant innovation. In addition, the SW, AMWCY activists, and Ecobank staff offered basic financial literacy training to interested C&Y, while the *Hope Point* functioned as a place of refuge. Although it was already known to and used by C&Y, it was hoped that SaVa could attract more of them to it, with the bank serving as an entry point for orientation towards other necessary services such as healthcare or school reinsertion.

Research into the pilot sought to understand whether, how, and for whom it 'worked', with which implications for child protection practice. Methods were mixed but lack the representativity of a Randomised Control Trial (RCT). C&Y researchers were trained to interview and use collective drawing with their peers, which they did on a bi-monthly basis. The SW recorded information on case management, training, and additional services offered. He also administered a survey to all C&Y at the point of entry into the SaVa project and after 1 year, asking basic questions such as about recent experiences of violence or theft. At the end of the pilot's 12 months, the author conducted open-ended interviews with C&Y using the bank (6), C&Y researchers who used the bank (2), project staff and steering committee partners (10), and child protection stakeholders (4). One focus group was also conducted with C&Y participants (10), alongside limited participant observation of where C&Y sleep and work. This broad dataset offers a solid base from which to interpret the project's findings. Full discussion of the project and its research methods can be found and freely accessed in the open-access evaluation [15].

Results

Financial Matters

Consultations with Togolese child protection actors established a target of working with 150 C&Y over the year of the pilot. In terms of accounts opened, this was exceeded by over 30%, with 200 accounts opened, and with the age range of account holders between 10 and 22 and the gender composition predominantly male (which reflects the wider demographics of the street-connected C&Y population in Lomé and across the region [2]). Total deposits surpassed 600,000 FCFA, close to 800 GBP, with withdrawals totalling around 440,000 FCFA. Almost 1,400 transactions took place, averaging around 400 FCFA per transaction, which is more than most C&Y spend on food in a day (see Figures 2-5 below).

Figures 2-5: Saver and Savings Data from SaVa Pilot

Source: SaVa Accounts Data

Although excitement was palpable at the existence of a bank 'just for them' and at the technology involved to run it (95% of users declared themselves 'happy' with it), C&Y did also report numerous difficulties in using it. Opening times – which were restricted to daylight hours – were often inconvenient, especially for C&Y busy in the day or making their money

Confidential: For Review Only

and wishing to deposit it at night. The lack of reliable internet access – on which the banking app depends – hampered various financial transactions. And the app itself, at the time relatively new, often crashed, or experienced issues that prevented the processing of transactions.

These obstacles make the project's positive financial numbers appear even more impressive. Qualitative data suggest that they owe much to the efforts of the SW, the volunteers, and the Ecobank staff, who conducted awareness-raising activities to promote the bank in the immediate vicinity of the *Hope Point* and further afield where street-connected, migrant and working C&Y were known to be concentrated. Football matches and music were also staged, whilst the promise of a (much-needed) free meal brought many in in the early days.

A Reduction in Violence

C&Y using the bank reported a drastic reduction in theft and violence over the course of the project's pilot year. Fully 100% of the C&Y involved in the project reported experiencing some form of recent violence when opening their accounts at the start. After six months, this was down to 25% and at the end of the 12 pilot months, it was down below 10% – a 90% reduction, as can be seen in Figure 6 below. Theft similarly plummeted. At the project's inception, the vast majority of participating C&Y reported having been robbed at some point over the preceding six months, often more than once. By contrast, at the end of the pilot period, 67% of respondents had been 'theft-free' for the past six months.

Figure *6: Reductions in Violence Amongst SaVa Participants*

Source: SaVa SW Survey

Qualitative data echo these findings – in multiple interviews, participants confirmed that they no longer faced theft nor themselves stle. 'On ne vole plus rien maintenant' was a frequent refrain in this regard, and that phrase's double-meaning is important here – it can, and should, translate to 'we are not stolen from anymore' *and* 'we do not steal anymore'. The Tdh SW explained the cycle thus: when C&Y quickly spend their money for fear of losing it later, they at times end up with nothing and then find themselves resorting to theft in order to be able to eat. Evidently, the ability to access reliable savings mechanisms interrupted this cycle.

Skills Development, Hope, and Safety

The opening of individual savings accounts presented the SW and *Hope Point* volunteers with an opportunity to offer C&Y trainings in financial literacy, numeracy, and budgeting. These were complimented by guidance on how to develop 'a life plan'. Data suggest that this orientation was positively experienced by C&Y, many of whom reported the project giving them skills, 'a sense of hope', and 'something to work for'. Importantly, the psychological function of saving appears to have mattered for many, in that it gave C&Y an anchor in the future, something safe to work towards or through, and thus a vehicle for their aspiration,

Confidential: For Review Only

which many translated into plans to accumulate enough savings to enter an apprenticeship. For others, as interview transcripts repeatedly made clear, it was the *Hope Point* itself that was the most important thing that the project offered, since it represented a place of refuge in lives that are hard, insecure, and often vulnerable:

'Here is somewhere we can come and rest, sleep, be safe…'
Interview with a 15-year-old Boy

'Here we can play, eat, hang out with our friends…'
15-year-old male Focus Group Participant

A Gateway for Other Services

As anticipated, the offer of digital micro-banking services to these C&Y acted as an entry point for providing other support and services. 54 C&Y, for example, were oriented towards social or health services according to their needs. Many benefited from interpersonal or family mediation. Some received empathy when they came to the *Hope Point* with their difficulties; others advice when they needed it. As Table 1 below makes clear, fully 111 C&Y went through situation-changing interventions during the life of the pilot, including 33 who left the streets.

Table 1: Secondary Services Provided

C&Y SUPPORTED THROUGH HOPE POINT			
**Target Group**	**Boys**	**Girls**	**Total**
Children put back in school	19	0	19
Children kept in school	08	07	15
Children taken out of work and put in an apprenticeship	13	06	19
Children supported to stay in an apprenticeship	02	06	08
Children taken off the streets and reunited with their families	33	17	50
**Totals**	**75**	**36**	**111**

Source: SaVa SW Data

Discussion

Evaluation of the SaVa pilot demonstrates, first and foremost, that it *is* possible to provide financial inclusion for street-connected C&Y – a population almost universally excluded from formal financial services. The SaVa model of partnering a bank and a child protection institution willing to act as the 'sponsor' complying with Know-Your-Customer legislation and then offering over-the-counter services appears unique. In this respect, it represents a potentially significant departure for C&Y financial inclusion, in particular for the highly vulnerable, typically marginalised group that is the street-connected.

Confidential: For Review Only

Second, the reductions in violence and theft associated with the pilot are undoubtedly impressive, but it is impossible to attribute causality entirely to the innovation of micro-banking alone. Indeed, it is scientifically possible that these results are just chance, while there is an evident risk of respondent bias, with C&Y answering questions with what they think those asking would like to hear. Yet qualitative research squarely point in the direction of alternative, more promising explanations. First, those depositing their money in the digital micro-bank appear to have become less attractive to potential thieves and claimed as much themselves. Second, having a savings account reduced the imperative for C&Y to quickly spend what money they have and thus end up resorting to theft themselves. Third, and most importantly, data suggest that the sensitisation and diligent on-the-ground work by the SW and his volunteers was paramount in shifting social norms amongst C&Y away from theft and violence. For although C&Y would previously be robbed by gangs of youths or predatory adults, they would often simply steal from each other, since such behaviour was normalised. In this respect, SaVa's digital micro-banking represented a kind of 'hook' around which more traditional, SW-led behaviour change work could take place.

Our third essential finding is that while micro-banking is attractive and impactful for C&Y, part of its power lies in bringing them into the protective 'net' and thus opening a pathway for the delivery of important, parallel services. Building on this, we may argue that while the evaluation methodology does not enable full disaggregation of the relative impacts on C&Y of SW support, the provision of a safe space, and the provision of banking services, this does not matter, since we can be highly confident that this *combination* is effective. Indeed, perhaps the major conclusion of our analysis must be that this combination 'works' for the vulnerable population in question. C&Y enjoyed having access to financial services and opening a savings account evidently supported the lengthening of their financial time horizons, which in turn had consequences for their ability to plan and aspire as well as to avoid quick spending and thus future theft. C&Y further enjoyed the safety and succour available at the *Hope Point* and the support provided by their SW, which included orientation to parallel services. In this respect, the study confirms that 'traditional' offers such as safe spaces and orientation to other services must continue to play a role in C&Y protection, but that digital micro-banking is a worthy addition to the protective mix. It further suggests that the effectiveness of digital micro-banking will depend on its being embedded in a traditional C&Y protection offering.

This study is evidently only a single case and a pilot at that; as such, its findings are necessarily limited. This is compounded by the fact that evaluation was not through an RCT, meaning that generalisability is impossible. Nevertheless, the findings are intriguing enough to suggest that replication and scale-up is worthwhile for the international child protection community, and that policymakers and practitioners would do well to replicate the SaVa model and conduct further research towards refinement.

References

[1] Committee on the Rights of the Child (CRC) (2017), *General comment No. 21 (2017) on children in street situations*, Geneva: Office for the Commission on Human Rights. Available at:

Confidential: For Review Only

https://tbinternet.ohchr.org/_layouts/15/treatybodyexternal/Download.aspx?symbolno=CRC%2fC%2fGC%2f21&Lang=en

[2] Howard, N., Jacquemin, M. & Thorsen, D. (2018), *Protecting Child Migrants Along the Abidjan-Lagos Corridor: Baseline Research Report*, Lausanne: Terre des Hommes Foundation. Available at: https://purehost.bath.ac.uk/ws/portalfiles/portal/203723196/26.pdf

[3] Amirali, A. (2018), *Child-Focused Micro Banks: Learning from Existing Programme Models*, Unpublished Report, Lausanne: Terre des Hommes Lausanne.

[4] Butterflies (2017), *My Khazana, My Dreams: A Book on Children's Experiences with Children's Development Khazana*, July 2017, New Delhi: Butterflies NGO.

[5] Zou, L., Tlapek, S., Njenga, G., Appiah, E., Opai-Tetteh, D. and Sherraden, M., (2015), 'Facilitators and Obstacles in Youth Saving: Perspectives from Ghana and Kenya', *Global Social Welfare*, 2.2: pp. 65–74.

[6] Lombe, M., & Ssewamala, F. M. (2007). 'The role of informal social networks in micro-savings mobilization'. *J. Soc. & Soc. Welfare*, 34, 37.

[7] Ssewamala, F. M., & Ismayilova, L. (2009). 'Integrating children's savings accounts in the care and support of orphaned adolescents in rural Uganda'. *Social Service Review*, 83(3), 453-472.

[8] Deshpande, R. and Zimmerman, J., 2010, *Youth Savings in Developing Countries: Trends in Practice, Gaps in Knowledge*, YouthSave Consortium, Washington D.C.

[9] Meagher, K., (2016), Conference Report, *Consolidating Africa's Mobile Banking Revolution*, Blavatnik School of Public Policy, University of Oxford.

[10] Flynn, J. and Sumberg, J., (2017), *Youth Savings Groups in Africa: They're a Family Affair*, Enterprise Development and Microfinance, 09/2017, Vol.28(3), pp. 147-161.

[11] Flynn, J. and Sumberg, J., (2018), *Savings Groups for Young Africans: A (Financial) Game Changer?*, Opinion, Institute of Development Studies (IDS). Accessible at: http://www.ids.ac.uk/opinion/savings-groups-for-young-africans-a-financial-game-changer.

[12] Karimli, L., Ssewamala, F. M., Neilands, T. B., & McKay, M. M. (2015). 'Matched child savings accounts in low-resource communities: who saves?'. *Global Social Welfare*, 2, 53-64.

[13] Karimli, L., & Ssewamala, F. M. (2015). 'Do savings mediate changes in adolescents' future orientation and health-related outcomes? Findings from randomized experiment in Uganda'. *Journal of Adolescent Health*, 57(4), 425-432.

[14] Yiga, D. and Raman, V., (2017), *Final Evaluation Report: Butterflies Investment Grant 2011-2017*, Kampala: Development Links Consulting Ltd.

Confidential: For Review Only

[15] Howard, N. (2020), *Pilot Evaluation Report: Sava - Safe Saving*, Lausanne: Terre des Hommes Foundation. Available at: https://purehost.bath.ac.uk/ws/portalfiles/portal/212996491/_SaVa_Pilot_Evaluation_Report.pdf

Figure Legends

Figure 1: *The Hope Point Drop-In Centre*
Figures 2-5: Saver and Savings Data from SaVa Pilot

Figure *6: Reductions in Violence Amongst SaVa Participants*

Confidential: For Review Only

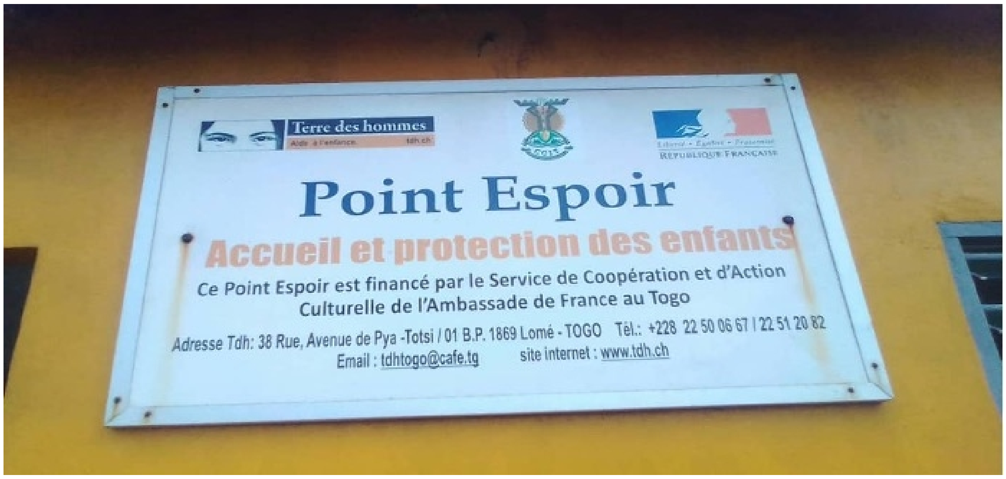

caption/legend in document

122x57mm (144 x 144 DPI)

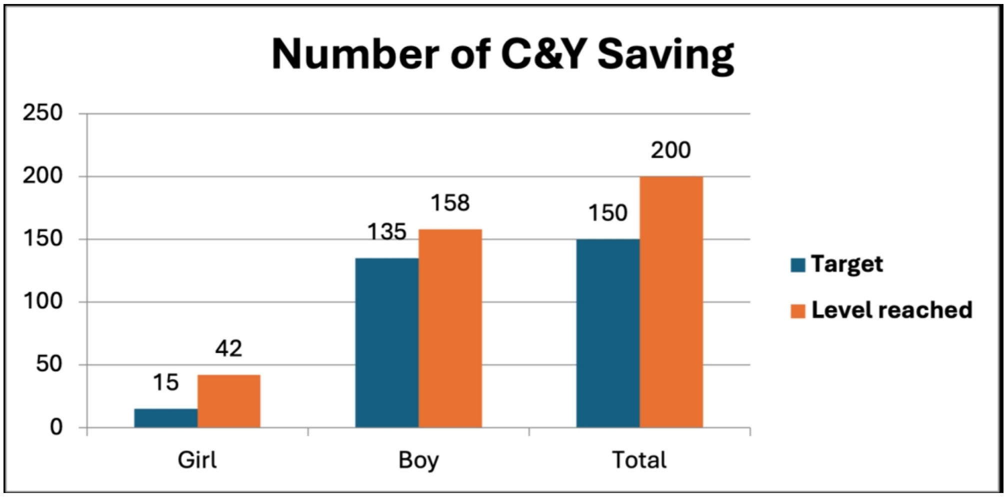

138x67mm (300 x 300 DPI)

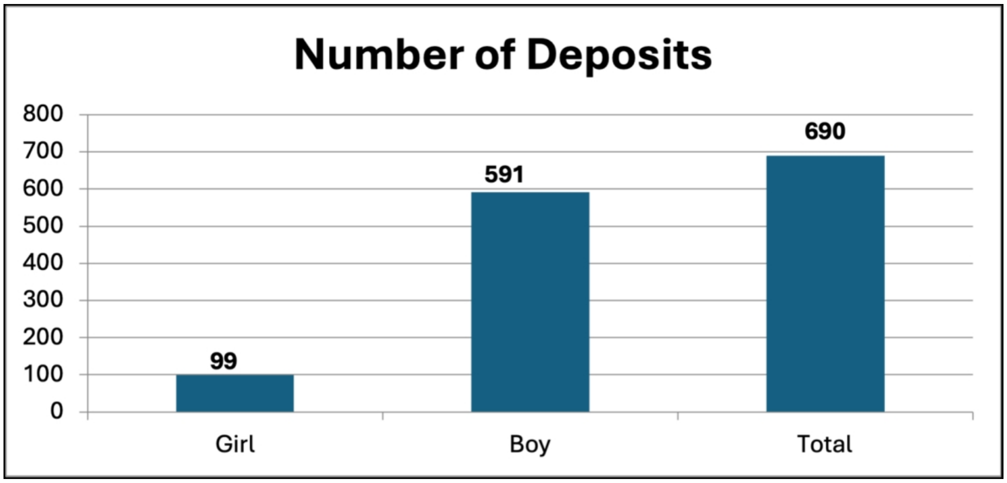

137x65mm (300 x 300 DPI)

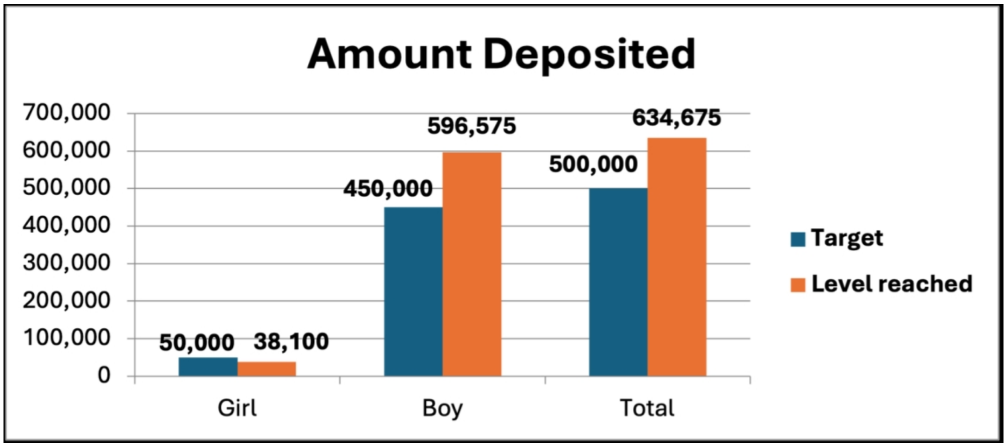

138x60mm (300 x 300 DPI)

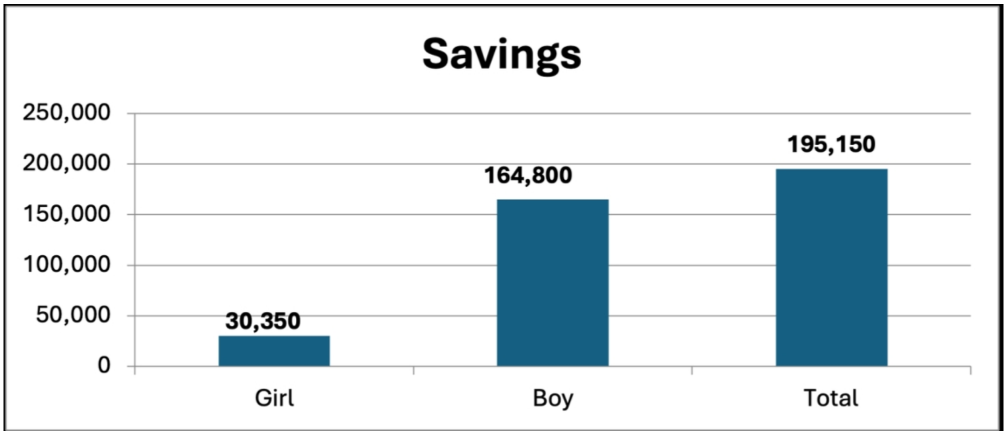

138x59mm (300 x 300 DPI)

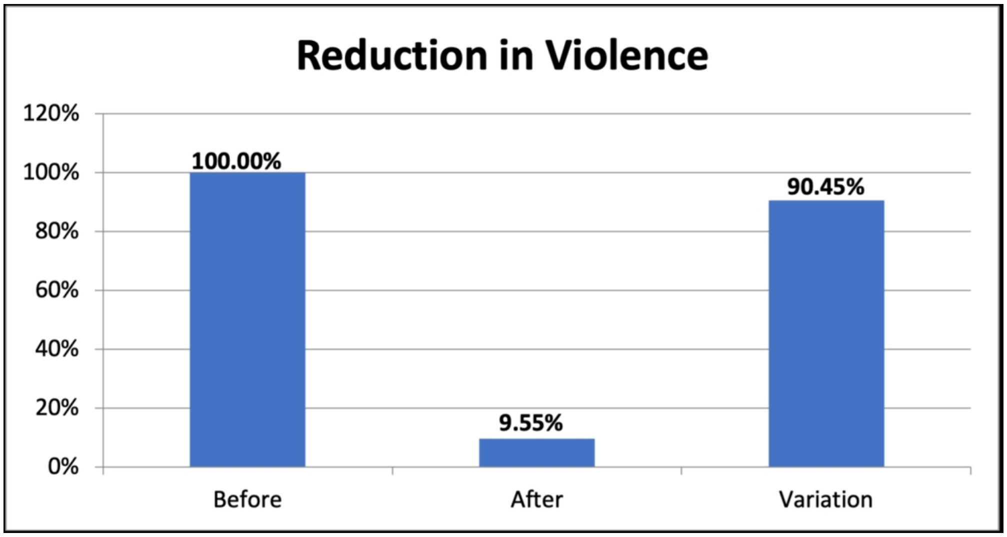

138x73mm (300 x 300 DPI)

BMJ Paediatrics Open

Digital Micro-Banking as a Health and Protection Intervention for Street-Connected Children and Youth? Analysis from a Togolese Pilot

(For consideration in the Special Collection on Health and Wellbeing of Street and Working Children, in collaboration with the International Society for Social Pediatrics and Child Health (ISSOP))

Journal:	*BMJ Paediatrics Open*
Manuscript ID	bmjpo-2023-002423.R2
Article Type:	Original research
Date Submitted by the Author:	20-Mar-2024
Complete List of Authors:	Howard, Neil; University of Bath Faculty of Humanities and Social Sciences, Social
Keywords:	Anthropology, Adolescent Health, Child Abuse, Social work

SCHOLARONE™
Manuscripts

I, the Submitting Author has the right to grant and does grant on behalf of all authors of the Work (as defined in the below author licence), an exclusive licence and/or a non-exclusive licence for contributions from authors who are: i) UK Crown employees; ii) where BMJ has agreed a CC-BY licence shall apply, and/or iii) in accordance with the terms applicable for US Federal Government officers or employees acting as part of their official duties; on a worldwide, perpetual, irrevocable, royalty-free basis to BMJ Publishing Group Ltd ("BMJ") its licensees and where the relevant Journal is co-owned by BMJ to the co-owners of the Journal, to publish the Work in this journal and any other BMJ products and to exploit all rights, as set out in our licence.

The Submitting Author accepts and understands that any supply made under these terms is made by BMJ to the Submitting Author unless you are acting as an employee on behalf of your employer or a postgraduate student of an affiliated institution which is paying any applicable article publishing charge ("APC") for Open Access articles. Where the Submitting Author wishes to make the Work available on an Open Access basis (and intends to pay the relevant APC), the terms of reuse of such Open Access shall be governed by a Creative Commons licence – details of these licences and which Creative Commons licence will apply to this Work are set out in our licence referred to above.

Other than as permitted in any relevant BMJ Author's Self Archiving Policies, I confirm this Work has not been accepted for publication elsewhere, is not being considered for publication elsewhere and does not duplicate material already published. I confirm all authors consent to publication of this Work and authorise the granting of this licence.

Confidential: For Review Only

Title Page

Title

Digital Micro-Banking as a Health and Protection Intervention for Street-Connected Children and Youth? Analysis from a Togolese Pilot

Corresponding Author

Neil Howard, PhD,
University of Bath
Claverton Down
BA2 7AY
UK
Email: nph28@bath.ac.uk

Key Words

Street-connected children and youth, child protection, violence against children, child banking

Word Count

2495

Acknowledgement

The author would like to acknowledge the contributions of the entire SaVa project team to the design and evaluation of the project.

Contributorship Statement

Dr. Howard is the sole author of this article.

Funding Statement

This work was supported by an Innovation Fund grant (Safe Savings – SaVa') from the international child protection organisation, Terre des Hommes Lausanne (Tdh): Avenue de Montchoisi 15, CH-1006, Lausanne, Switzerland. It partnered Tdh, Ecobank, the African Movement of Working Children and Youth (AMWCY), and the University of Bath (UoB).

Confidential: For Review Only

Ethics Approval statements

This project was granted ethical approval after internal review by Tdh's project ethics advisor on Dec 1 2018. It received approval by the University of Bath's Social Science Research Ethics Committee, with number 2670-3099 2670-3099.

Competing Interests Statement

The author completed the ICMJE uniform disclosure form at http://www.icmje.org/disclosure-of-interest/ and declares: I received funding from Terre des Hommes to lead the team conducting the action research that led to the proposal of the pilot studied in this paper; I further received funding to participate in design and evaluation of the pilot. This funding ended in 2020.

Patient and Public Involvement Statement

Members of the public were involved as research participants at every stage of the project reported on. The intervention design was rooted in a participatory action research project that consulted some of the children and youth (C&Y) who would eventually participate in the intervention and its evaluation (cited below as Howard et al. 2018). C&Y researchers were recruited and trained to gather and analyse data; initial findings were shared and cross-checked with them. Interviews with C&Y were open-ended and left space for C&Y to guide them. Fun, creative methods were also used.

Data Sharing Statement

The unpublished report behind this paper can be freely downloaded at https://purehost.bath.ac.uk/ws/portalfiles/portal/212996491/_SaVa_Pilot_Evaluation_Report.pdf. Interview schedules and some anonymised primary data may be shared upon request; others cannot be shared for data protection reasons.

Confidential: For Review Only

Main Document

Abstract

Background:

The SaVa pilot offered street-connected children and youth (C&Y) access to digital micro-banking services to help them save money and thus avoid the violence associated with theft at night. It further used the micro-banking service as a gateway to attract C&Y towards other health and protection services. It took place over 12 months in Lomé, Togo, and involved one social worker (SW) plus volunteers running 'the bank' in an NGO-funded drop-in centre.

Methods:

C&Y peer researchers were trained to interview and use collective drawing with peers, which they did on a bi-monthly basis. The SW recorded information about case management, training, and additional services offered. He administered a survey to C&Y at the point of entry into the project and after 1 year, asking questions about recent experiences of violence or theft. At the end of 12 months, the author conducted interviews with C&Y using the bank, C&Y researchers who used the bank, project staff and steering committee partners, and child protection stakeholders. One focus group was conducted with C&Y participants, alongside limited participant observation of where C&Y sleep and work.

Results:

Findings suggest that the intervention was successful in encouraging children to open accounts and save money, and in contributing to a reduction in theft and associated violence. The incidence of theft reduced 90%; 95% of C&Y account holders were happy with the service; 200 accounts were opened. The project supported the delivery of additional services, including counselling, school reinsertion, and removal from the streets.

Conclusions:

Analysis suggests that it is possible to offer street-connected C&Y formal financial services but that that this offer has potential as a health and protection intervention, especially in combination with the provision of a safe space, dedicated SW support, and add-on services.

Key Message

- **What is already known on this topic**

There are millions of street-connected C&Y all around the world and they are known to be vulnerable to theft and associated violence. Lacking access to formal financial services, they

Confidential: For Review Only

typically rely on informal and often unreliable savings mechanisms which deepen their vulnerability.

- **What this study adds**

This study shows that with the right institutional accompaniment, digital micro-banking is possible for street-connected C&Y and that it can contribute to increased savings and reduced theft and associated violence. It further shows that digital micro-banking can be used as an attractive 'hook' to bring C&Y into the orbit of additional services.

- **How this study might affect research, practice or policy**

The findings strongly suggest that child protection actors should consider incorporating the offer of digital micro-banking services alongside more traditional offers of safe spaces, SW support, and service referral.

Key Findings

There are millions of street-connected C&Y all around the world and they are known to be vulnerable to theft and associated violence. Lacking access to formal financial services, they typically rely on informal and often unreliable savings mechanisms which deepen their vulnerability. This study shows that with the right institutional accompaniment, digital micro-banking is possible for street-connected C&Y and that it can contribute to increased savings and reduced theft and associated violence. It further shows that digital micro-banking can be used as an attractive 'hook' to bring C&Y into the orbit of additional services. The findings strongly suggest that child protection actors should consider incorporating the offer of digital micro-banking services alongside more traditional offers of safe spaces, SW support, and service referral.

Introduction

There are millions of street-connected C&Y around the world and they are known to be vulnerable to violence, including theft and associated assault [1]. In 2017, the author led a participatory action research study with street-connected migrant and working C&Y in five West African cities, which found that theft, especially at night, was among the greatest issues they faced [2]. Not only was theft traumatic and often accompanied by violence, but the fact that C&Y were so frequently robbed meant that they were effectively stuck in a poverty trap, unable to accumulate enough money to get off the streets or to go home. This dynamic is well-established in the literature, which shows that C&Y use multiple, often quite ingenious, informal avenues for saving – ranging from entrusting their money to a local adult to burying it overnight – but that none of these remain reliable in the long-term [3-7]. The literature further emphasises that there are substantial legal and cultural barriers to offering street-connected C&Y formal financial services. Lacking a fixed address or formal identification documents, few street-connected C&Y can meet the legal Know-Your-Customer requirements underpinning most formal banking, including mobile money banking, while informal barriers such as discrimination on the part of bank staff or shame on the part of C&Y

Confidential: For Review Only

are also impactful [3, 8]. Although there is some precedent for C&Y savings groups in Africa [9-13] and for pseudo-banking services for street-connected C&Y in South Asia [4, 14], the project's benchmarking study found no evidence either of the widespread provision of formal financial services to street-connected C&Y or of the use of digital services to this end [3]. Consequently, C&Y remain excluded from financial services and more likely to face theft, violence, and the ongoing cycle of poverty.

The SaVa pilot emerged in this context and was implemented by the international child protection organisation, Terre des Hommes (Tdh). It sought to examine the theft- and violence-reduction potential of providing these street-connected C&Y with access to digital micro-banking services in combination with more 'traditional' support such as a safe space, SW accompaniment, and referral. The hypothesis was that if C&Y had somewhere safe and accessible to put their money, they would be less likely to keep it on their person and thus less vulnerable to theft, whilst also being less likely to avail themselves of parallel informal savings mechanisms that often let them down. We also anticipated that children would be attracted to the technology of the micro-banking service, which would in turn act as a 'gateway' for additional child protection services. The research sought to establish the validity or otherwise of these expectations[1].

Methods

The SaVa model was straightforward. Operationally, it centred on the Tdh *Hope Point* at the Aflao border with Ghana. *Hope Points* are safe spaces for C&Y to come and rest, play, or receive SW support (see Figure 1 below). This one served the street-connected population based around Lomé beach and was staffed by a senior SW. He was supported on a rotating basis by four experienced activists from the African Movement for Working Children and Youth (AMWCY), who did outreach work and contributed to the management of micro-banking services.

Figure 1: The Hope Point Drop-In Centre

© Author – Permission Granted for Use

Day-to-day, the *Hope Point* operated like a bank, with C&Y coming to deposit and withdraw money during opening hours. Banking was provided by Ecobank in the form of an *Xpress Point*, which is an Ecobank-approved, sub-contracted mobile money service. This bank included a counter, secured smartphones for transactions, and SIM cards for each individual account, which were kept in the *Hope Point* overnight to prevent loss or theft. Crucially, while Ecobank provided the financial infrastructure, Tdh acted as the legal sponsor for each account – in other words, each mobile money account and the SIM card associated with it was opened in Tdh's name (so as to enable the project and the bank to comply with Know-Your-Customer requirements), but Tdh kept a ledger in the *Hope Point* linking each SIM to their eventual C&Y 'owner'. This was a significant innovation. In addition, the SW, AMWCY activists, and Ecobank staff offered basic financial literacy training to interested C&Y, while the *Hope Point* functioned as a place of refuge. Although it was already known to and used by C&Y, it was

[1] Parts of this paper draw on and reproduce text from the project's evaluation [15].

Confidential: For Review Only

hoped that SaVa could attract more of them to it, with the bank serving as an entry point for orientation towards other necessary services such as healthcare or school reinsertion.

Research into the pilot sought to understand whether, how, and for whom it 'worked', with which implications for child protection practice. Methods were mixed but lack the representativity of a Randomised Control Trial (RCT). C&Y researchers were trained to interview and use collective drawing with their peers, which they did on a bi-monthly basis. The SW recorded information on case management, training, and additional services offered. He also administered a survey to all C&Y at the point of entry into the SaVa project and after 1 year, asking basic questions such as about recent experiences of violence or theft. At the end of the pilot's 12 months, the author conducted open-ended interviews with C&Y using the bank (6), C&Y researchers who used the bank (2), project staff and steering committee partners (10), and child protection stakeholders (4). One focus group was also conducted with C&Y participants (10), alongside limited participant observation of where C&Y sleep and work. This broad dataset offers a solid base from which to interpret the project's findings. Full discussion of the project and its research methods can be found and freely accessed in the open-access evaluation [15].

Results

Financial Matters

Consultations with Togolese child protection actors established a target of working with 150 C&Y over the year of the pilot. In terms of accounts opened, this was exceeded by over 30%, with 200 accounts opened, and with the age range of account holders between 10 and 22 and the gender composition predominantly male (which reflects the wider demographics of the street-connected C&Y population in Lomé and across the region [2]). Total deposits surpassed 600,000 FCFA, close to 800 GBP, with withdrawals totalling around 440,000 FCFA. Almost 1,400 transactions took place, averaging around 400 FCFA per transaction, which is more than most C&Y spend on food in a day (see Figures 2-5 below).

Figures 2-5: Saver and Savings Data from SaVa Pilot

Source: SaVa Accounts Data

Although excitement was palpable at the existence of a bank 'just for them' and at the technology involved to run it (95% of users declared themselves 'happy' with it), C&Y did also report numerous difficulties in using it. Opening times – which were restricted to daylight hours – were often inconvenient, especially for C&Y busy in the day or making their money and wishing to deposit it at night. The lack of reliable internet access – on which the banking app depends – hampered various financial transactions. And the app itself, at the time relatively new, often crashed, or experienced issues that prevented the processing of transactions.

These obstacles make the project's positive financial numbers appear even more impressive. Qualitative data suggest that they owe much to the efforts of the SW, the volunteers, and the

Confidential: For Review Only

Ecobank staff, who conducted awareness-raising activities to promote the bank in the immediate vicinity of the *Hope Point* and further afield where street-connected, migrant and working C&Y were known to be concentrated. Football matches and music were also staged, whilst the promise of a (much-needed) free meal brought many in in the early days.

A Reduction in Violence

C&Y using the bank reported a drastic reduction in theft and violence over the course of the project's pilot year. Fully 100% of the C&Y involved in the project reported experiencing some form of recent violence when opening their accounts at the start. After six months, this was down to 25% and at the end of the 12 pilot months, it was down below 10% – a 90% reduction, as can be seen in Figure 6 below. Theft similarly plummeted. At the project's inception, the vast majority of participating C&Y reported having been robbed at some point over the preceding six months, often more than once. By contrast, at the end of the pilot period, 67% of respondents had been 'theft-free' for the past six months.

Figure *6: Reductions in Violence Amongst SaVa Participants*

Source: SaVa SW Survey

Qualitative data echo these findings – in multiple interviews, participants confirmed that they no longer faced theft nor themselves stle. 'On ne vole plus rien maintenant' was a frequent refrain in this regard, and that phrase's double-meaning is important here – it can, and should, translate to 'we are not stolen from anymore' *and* 'we do not steal anymore'. The Tdh SW explained the cycle thus: when C&Y quickly spend their money for fear of losing it later, they at times end up with nothing and then find themselves resorting to theft in order to be able to eat. Evidently, the ability to access reliable savings mechanisms interrupted this cycle.

Skills Development, Hope, and Safety

The opening of individual savings accounts presented the SW and *Hope Point* volunteers with an opportunity to offer C&Y trainings in financial literacy, numeracy, and budgeting. These were complimented by guidance on how to develop 'a life plan'. Data suggest that this orientation was positively experienced by C&Y, many of whom reported the project giving them skills, 'a sense of hope', and 'something to work for'. Importantly, the psychological function of saving appears to have mattered for many, in that it gave C&Y an anchor in the future, something safe to work towards or through, and thus a vehicle for their aspiration, which many translated into plans to accumulate enough savings to enter an apprenticeship. For others, as interview transcripts repeatedly made clear, it was the *Hope Point* itself that was the most important thing that the project offered, since it represented a place of refuge in lives that are hard, insecure, and often vulnerable:

> 'Here is somewhere we can come and rest, sleep, be safe…'
> *Interview with a 15-year-old Boy*

Confidential: For Review Only

'Here we can play, eat, hang out with our friends…'
15-year-old male Focus Group Participant

A Gateway for Other Services

As anticipated, the offer of digital micro-banking services to these C&Y acted as an entry point for providing other support and services. 54 C&Y, for example, were oriented towards social or health services according to their needs. Many benefited from interpersonal or family mediation. Some received empathy when they came to the *Hope Point* with their difficulties; others advice when they needed it. As Table 1 below makes clear, fully 111 C&Y went through situation-changing interventions during the life of the pilot, including 33 who left the streets.

Table 1: Secondary Services Provided

C&Y SUPPORTED THROUGH HOPE POINT			
**Target Group**	**Boys**	**Girls**	**Total**
Children put back in school	19	0	19
Children kept in school	08	07	15
Children taken out of work and put in an apprenticeship	13	06	19
Children supported to stay in an apprenticeship	02	06	08
Children taken off the streets and reunited with their families	33	17	50
**Totals**	**75**	**36**	**111**

Source: SaVa SW Data

Discussion

Evaluation of the SaVa pilot demonstrates, first and foremost, that it *is* possible to provide financial inclusion for street-connected C&Y – a population almost universally excluded from formal financial services. The SaVa model of partnering a bank and a child protection institution willing to act as the 'sponsor' complying with Know-Your-Customer legislation and then offering over-the-counter services appears unique. In this respect, it represents a potentially significant departure for C&Y financial inclusion, in particular for the highly vulnerable, typically marginalised group that is the street-connected.

Second, the reductions in violence and theft associated with the pilot are undoubtedly impressive, but it is impossible to attribute causality entirely to the innovation of micro-banking alone. Indeed, it is scientifically possible that these results are just chance, while there is an evident risk of respondent bias, with C&Y answering questions with what they think those asking would like to hear. Yet qualitative research squarely point in the direction of alternative, more promising explanations. First, those depositing their money in the digital micro-bank appear to have become less attractive to potential thieves and claimed as much

Confidential: For Review Only

themselves. Second, having a savings account reduced the imperative for C&Y to quickly spend what money they have and thus end up resorting to theft themselves. Third, and most importantly, data suggest that the sensitisation and diligent on-the-ground work by the SW and his volunteers was paramount in shifting social norms amongst C&Y away from theft and violence. For although C&Y would previously be robbed by gangs of youths or predatory adults, they would often simply steal from each other, since such behaviour was normalised. In this respect, SaVa's digital micro-banking represented a kind of 'hook' around which more traditional, SW-led behaviour change work could take place.

Our third essential finding is that while micro-banking is attractive and impactful for C&Y, part of its power lies in bringing them into the protective 'net' and thus opening a pathway for the delivery of important, parallel services. Building on this, we may argue that while the evaluation methodology does not enable full disaggregation of the relative impacts on C&Y of SW support, the provision of a safe space, and the provision of banking services, this does not matter, since we can be highly confident that this *combination* is effective. Indeed, perhaps the major conclusion of our analysis must be that this combination 'works' for the vulnerable population in question. C&Y enjoyed having access to financial services and opening a savings account evidently supported the lengthening of their financial time horizons, which in turn had consequences for their ability to plan and aspire as well as to avoid quick spending and thus future theft. C&Y further enjoyed the safety and succour available at the *Hope Point* and the support provided by their SW, which included orientation to parallel services. In this respect, the study confirms that 'traditional' offers such as safe spaces and orientation to other services must continue to play a role in C&Y protection, but that digital micro-banking is a worthy addition to the protective mix. It further suggests that the effectiveness of digital micro-banking will depend on its being embedded in a traditional C&Y protection offering.

This study is evidently only a single case and a pilot at that; as such, its findings are necessarily limited. This is compounded by the fact that evaluation was not through an RCT, meaning that generalisability is impossible. Nevertheless, the findings are intriguing enough to suggest that digital micro-banking *is* possible for street-connected C&Y and that it can contribute to increased savings and reduced theft and associated violence. They further show that digital micro-banking can be used as an attractive 'hook' to bring C&Y into the orbit of additional services. The findings suggest that child protection actors should consider incorporating this offer alongside more traditional offers of safe spaces, SW support, and service referral. The international child protection community would do well to replicate the SaVa model and conduct further research towards refinement.

References

[1] Committee on the Rights of the Child (CRC) (2017), *General comment No. 21 (2017) on children in street situations*, Geneva: Office for the Commission on Human Rights. Available at: https://tbinternet.ohchr.org/_layouts/15/treatybodyexternal/Download.aspx?symbolno=CRC%2fC%2fGC%2f21&Lang=en

Confidential: For Review Only

[2] Howard, N., Jacquemin, M. & Thorsen, D. (2018), *Protecting Child Migrants Along the Abidjan-Lagos Corridor: Baseline Research Report*, Lausanne: Terre des Hommes Foundation. Available at: https://purehost.bath.ac.uk/ws/portalfiles/portal/203723196/26.pdf

[3] Amirali, A. (2018), *Child-Focused Micro Banks: Learning from Existing Programme Models*, Unpublished Report, Lausanne: Terre des Hommes Lausanne.

[4] Butterflies (2017), *My Khazana, My Dreams: A Book on Children's Experiences with Children's Development Khazana*, July 2017, New Delhi: Butterflies NGO.

[5] Zou, L., Tlapek, S., Njenga, G., Appiah, E., Opai-Tetteh, D. and Sherraden, M., (2015), 'Facilitators and Obstacles in Youth Saving: Perspectives from Ghana and Kenya', *Global Social Welfare*, 2.2: pp. 65–74.

[6] Lombe, M., & Ssewamala, F. M. (2007). 'The role of informal social networks in micro-savings mobilization'. *J. Soc. & Soc. Welfare*, 34, 37.

[7] Ssewamala, F. M., & Ismayilova, L. (2009). 'Integrating children's savings accounts in the care and support of orphaned adolescents in rural Uganda'. *Social Service Review*, 83(3), 453-472.

[8] Deshpande, R. and Zimmerman, J., 2010, *Youth Savings in Developing Countries: Trends in Practice, Gaps in Knowledge*, YouthSave Consortium, Washington D.C.

[9] Meagher, K., (2016), Conference Report, *Consolidating Africa's Mobile Banking Revolution*, Blavatnik School of Public Policy, University of Oxford.

[10] Flynn, J. and Sumberg, J., (2017), *Youth Savings Groups in Africa: They're a Family Affair*, Enterprise Development and Microfinance, 09/2017, Vol.28(3), pp. 147-161.

[11] Flynn, J. and Sumberg, J., (2018), *Savings Groups for Young Africans: A (Financial) Game Changer*?**,** Opinion, Institute of Development Studies (IDS). Accessible at: http://www.ids.ac.uk/opinion/savings-groups-for-young-africans-a-financial-game-changer.

[12] Karimli, L., Ssewamala, F. M., Neilands, T. B., & McKay, M. M. (2015). 'Matched child savings accounts in low-resource communities: who saves?'. *Global Social Welfare*, 2, 53-64.

[13] Karimli, L., & Ssewamala, F. M. (2015). 'Do savings mediate changes in adolescents' future orientation and health-related outcomes? Findings from randomized experiment in Uganda'. *Journal of Adolescent Health*, 57(4), 425-432.

[14] Yiga, D. and Raman, V., (2017), *Final Evaluation Report: Butterflies Investment Grant 2011-2017*, Kampala: Development Links Consulting Ltd.

[15] Howard, N. (2020), *Pilot Evaluation Report: Sava - Safe Saving*, Lausanne: Terre des Hommes Foundation. Available at:

Confidential: For Review Only

https://purehost.bath.ac.uk/ws/portalfiles/portal/212996491/_SaVa_Pilot_Evaluation_Report.pdf

Figure Legends

Figure 1: *The Hope Point Drop-In Centre*
Figures 2-5: Saver and Savings Data from SaVa Pilot

Figure *6: Reductions in Violence Amongst SaVa Participants*

Confidential: For Review Only

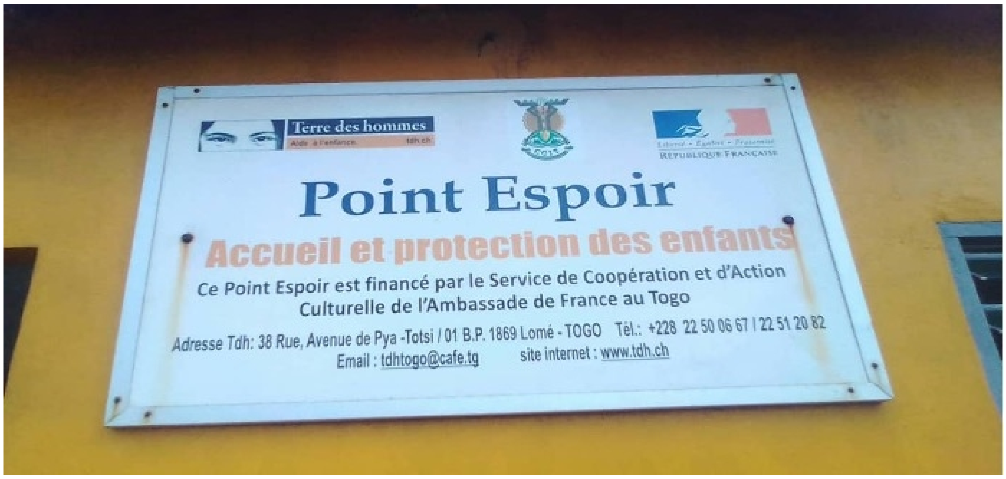

Figure 1: The Hope Point Drop-In Centre

122x57mm (144 x 144 DPI)

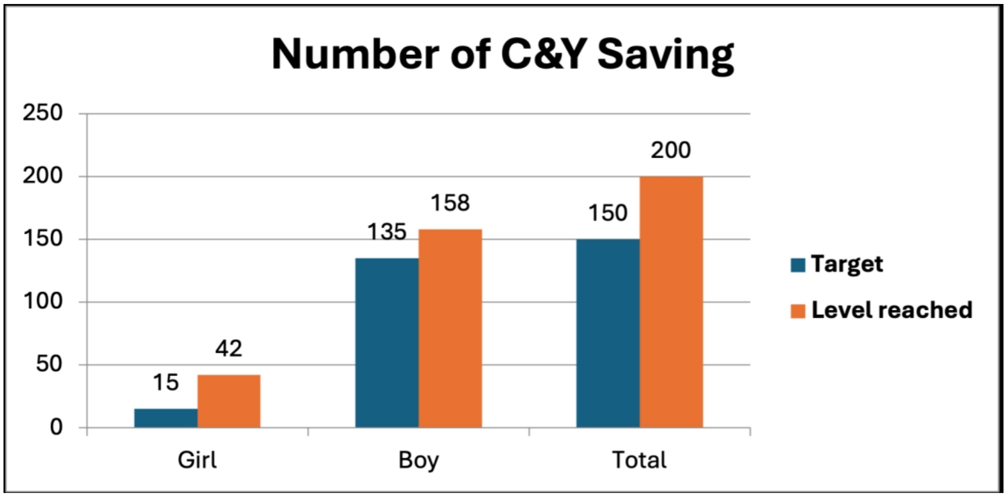

Figure 2 – Number of C&Y Saving

138x67mm (330 x 330 DPI)

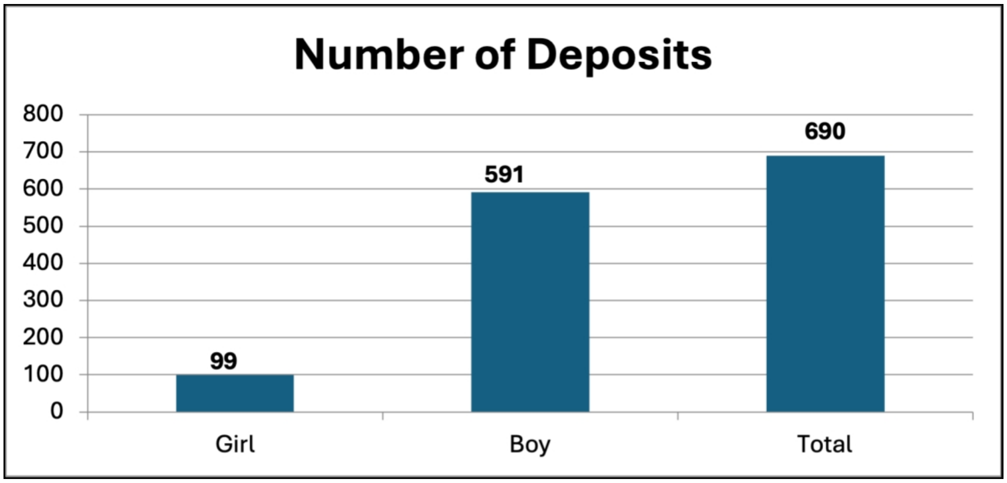

Figure 3 – Number of Deposits

137x65mm (330 x 330 DPI)

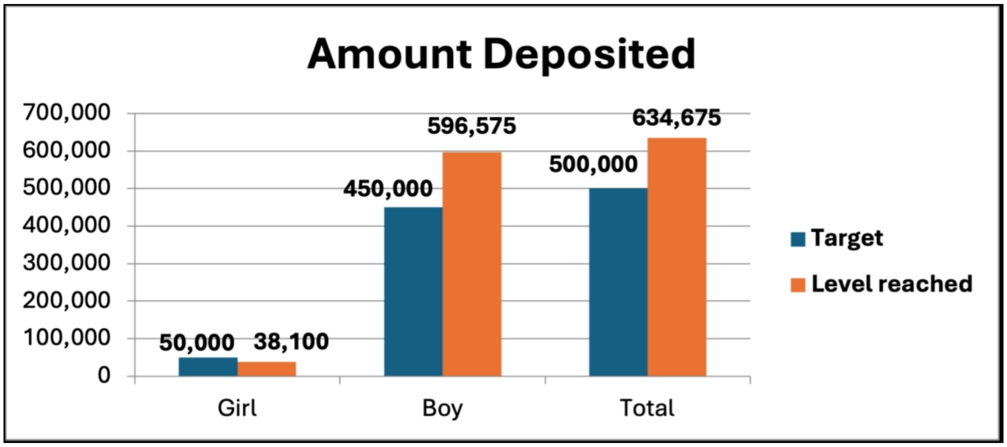

Figure 4 – Amount Deposited

138x60mm (330 x 330 DPI)

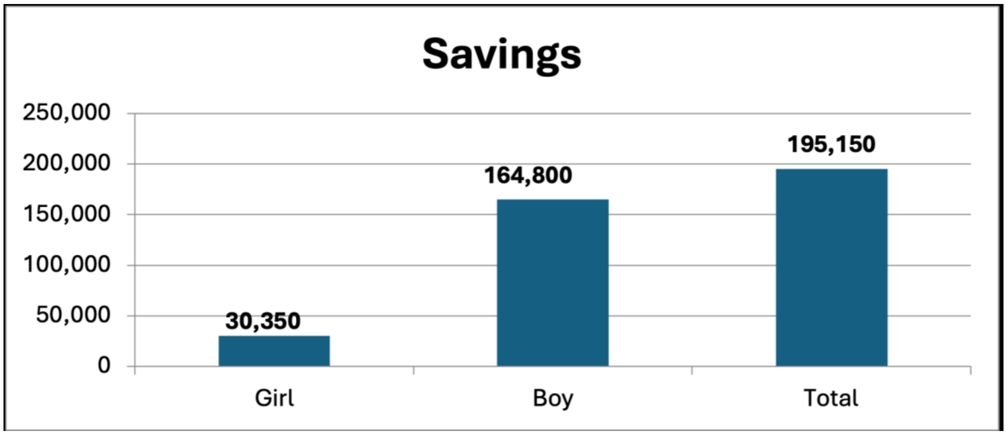

Figure 5 – Total Savings

138x59mm (330 x 330 DPI)

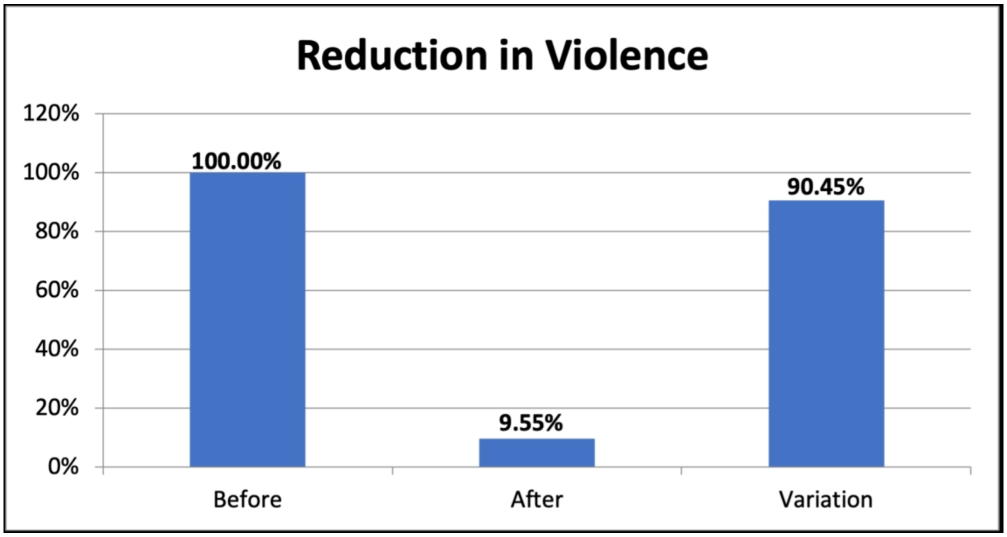

Figure 6: Reductions in Violence Amongst SaVa Participants

138x73mm (330 x 330 DPI)
